# Multiple examinations indicated associations between abnormal regional homogeneity and cognitive dysfunction in major depressive disorder

**DOI:** 10.3389/fpsyg.2022.1090181

**Published:** 2023-01-26

**Authors:** Yun Wang, Xiaoling Li, Haohao Yan, Qinqin Zhang, Yangpan Ou, Weibin Wu, Webo Shangguan, Wensheng Chen, Yang Yu, Jiaquan Liang, Wanting Wu, Hairong Liao, Zishan Liu, Xiancong Mai, Guojun Xie, Wenbin Guo

**Affiliations:** ^1^Department of Psychiatry, The Third People's Hospital of Foshan, Foshan, Guangdong, China; ^2^Department of Psychiatry, National Clinical Research Center for Mental Disorders, The Second Xiangya Hospital of Central South University, Changsha, Hunan, China

**Keywords:** major depressive disorder, regional homogeneity, support vector machine, salience network, cognition

## Abstract

**Background:**

This study aimed to investigate the relationships between regional neural activity and multiple related indicators in patients with major depressive disorder (MDD).

**Methods:**

Forty-two patients and 42 healthy controls (HCs) were enrolled. Pearson/Spearman correlation analyses were applied to examine the associations between abnormal regional homogeneity (ReHo) and different indicators in the patients.

**Results:**

Compared with HCs, patients with MDD had increased ReHo in the left inferior temporal gyrus (ITG) and decreased ReHo values in the left putamen, anterior cingulate cortex (ACC), and precentral gyrus. The ReHo of the left putamen was positively correlated with the PR interval, Repeatable Battery for the Assessment of Neuropsychological Status 4A, and Discriminant analysis (D), and negatively correlated with Ae (block) and Ae (total) in the patients. The ReHo value of the left ACC was positively correlated with the severity of depression, Stroop Color Word Test of C − 2B + 100 in reaction time, and negatively correlated with Ce (Missay) and Perseverative Responses in the patients. The ReHo of the left ITG was positively correlated with the Neuroticism scores and negatively correlated with the Lie scores in the patients.

**Conclusion:**

These results suggested that the decreased ReHo of the salience network might be the underpinning of cognitive impairments in patients with MDD.

## Introduction

Major depressive disorder (MDD) is a highly prevailing and disabling mental disorder associated with high morbidity and mortality (Fagiolini et al., 2013). About 20 to 30% of individuals with MDD evolve into chronic disease (Angst et al., 2009; Murphy and Byrne, 2012). It affects about 350 million people around the world. By 2030, MDD will be the leading cause of the burden of disease worldwide (Collins et al., 2011). Plenty of studies have shown that patients with MDD have aberrant brain imaging (Zhang et al., 2018; Liang et al., 2020; Yang et al., 2021), clinical (Caldwell and Steffen, 2018; Kircanski et al., 2019), event-related potentials (ERPs; Landes et al., 2018; Kim et al., 2020; Fan et al., 2021; Xin et al., 2021), and cognitive indicators (Pan et al., 2019). MDD is a heterogeneous disease (Harald and Gordon, 2012), and its etiology remains vague. Combining clinical factors, ERPs, cognitive indicators and other factors to explore the changes of brain functional activity in patients with MDD may provide valuable evidence for understanding the etiology of depression.

MDD is a psychological abnormality concerning mood dysregulation, including neuroendocrine changes, autonomic nervous system (ANS) disorder, immune system activation rhythm disturbances, and oxidative stress (Halaris, 2017). The Hamilton Depression Rating Scale (HAMD; Gibbons et al., 1993; Stefanis and Stefanis, 2001) and Hamilton Anxiety Rating Scale (HAMA; Clark and Donovan, 1994) have been frequently used to assess the severity of depression and anxiety separately to help clarify clinical diagnosis and degree division of mood disorders. The social disability screening schedule (SDSS; Yang et al., 2013), social support rating scale (SSRS; Qin et al., 2021), simplified coping style questionnaire (SCSQ; Lin et al., 2020), and Eysenck personality questionnaire (EPQ) are widely used to assess the psychological status of patients with MDD. Studies have shown that patients with MDD have abnormal EPQ scores (Małyszczak et al., 2019; Wu et al., 2019; Otsubo et al., 2021). Many investigations have reported that patients with MDD exhibit a variety of abnormal biological indexes during outpatient and hospitalization. Heart rate in patients with MDD is significantly increased at rest (Williams et al., 2011). The co-morbidity between MDD and cardiovascular disease (CVD) is a matter of public knowledge. A meta-analytic study found that MDD was recognized as a major risk element for mortality in coronary heart disease (CHD; Barth et al., 2004). And heart rate variability(HRV)pertains to the variation in heart rate and measures the interplay between the sympathetic and parasympathetic nervous systems (Kidwell and Ellenbroek, 2018). Previous researches have established the key element in the link between MDD and CVD with a declining HRV (Musselman et al., 1998; Stapelberg et al., 2012). Several meta-analysis studies have reported that MDD is associated with increased total triglyceride (TG) and cholesterol (CHOL), and low high density lipoprotein (HDL), low density lipoprotein (LDL), and omega-3 polyunsaturated fatty acids (PUFA; Lin et al., 2010; Pan et al., 2012; Persons and Fiedorowicz, 2016). Besides, the thyroid hormones in patients with MDD were lower than those in HCs (Zhou Y. et al., 2021), and thyroid hormones were applied as a supplementary treatment for MDD (Mcintyre, 2016). Moreover, decreased antioxidant uric acid was observed in patients with MDD (Bartoli et al., 2018). A large number of studies have described the relationship between Hypercortisolemia and MDD (Parker et al., 2003; Stetler and Miller, 2011; Nobis et al., 2020). Hypercortisolemia has been linked to depression with ruminations (Stewart et al., 2013), psychogenic depression (Schatzberg et al., 1984), and melancholic and psychotic depressive subtypes (Krishnan and Nestler, 2008; Kunugi et al., 2015). Hypercortisolemia was also depicted as a possible biomarker for treatment-resistant depression (TRD) and persisted after remission (Markopoulou et al., 2009).

A systematic review and meta-analysis by Rock and Roiser et al. suggested that cognitive impairment was a central feature of MDD (Rock et al., 2014). About two-thirds of patients with MDD have cognitive impairment (Afridi et al., 2011). Studies have shown that cognitive impairment persists beyond the acute episode of MDD, with a third to half of the remissive patients with MDD still having a cognitive impairment (Reppermund et al., 2009).

Notably, There is a good deal of tools widely used in measuring cognitive function in MDD, including the Wisconsin Card sorting test (WCST; Nakano et al., 2008; Liao et al., 2021), Stroop Color Word Test (SCWT; Shi et al., 2020; Duan et al., 2021; Zhou Q. et al., 2021), Repeatable Battery for the Assessment of Neuropsychological Status (RBANS; Guan et al., 2021; Teng et al., 2021), eye-tracking test (Takahashi et al., 2021), and ERP (Bruder et al., 2011; Jaworska and Protzner, 2013; Klawohn et al., 2021; Tseng et al., 2021; White et al., 2021), which have proved that the cognitive domains including executive functioning, learning and memory, processing speed, decision-making, concentration, and attention are notably impaired in patients with MDD (Pan et al., 2019; Wang et al., 2020).

Most of the research theories on the causes of MDD comprise of psychological, biological, and social factors (Beck and Bredemeier, 2016; Brouwer et al., 2019), but few studies have combined these factors in a single study (Kennis et al., 2019). Many of them mainly discuss the relationship between a certain index of patients with MDD and the whole brain activity, whereas there are few studies on exploring local brain activity in patients with MDD by combining multiple indicators. Brain activity in the bilateral orbital frontal cortex (OFC) of patients with MDD was reduced, resulting in decreased ability of patients with MDD to inhibit negative stimuli (Zhang et al., 2016). A structural neuroimaging study has shown decreased volume of the caudate nucleus and lateral orbitofrontal cortex (LOC) and a thinning of the bilateral insula cortex, which are associated with decreased working memory performance and processing speed in MDD (Saleh et al., 2017). The dynamic functional network connectivity in MDD showed that stronger links existed among sensory-related regions than those in HCs (HCs), which were connected with extroversion and neuroticism of the EPQ in the patients (Wu et al., 2019). A study combining ERP and functional magnetic resonance imaging(fMRI) showed that ventral striatum activation and feedback negativity amplitude decreased in patients with MDD, and there was a significant correlation between feedback negativity amplitude and ventral striatum activation (Foti et al., 2014). Hence, it has a pivotal role in discussing the correlation between brain function changes and functional impairment in patients with MDD.

Regional Homogeneity (ReHo) is a voxel-based analysis, which is according to the synchronicity between the time sequences of a given voxel and its neighboring voxels. ReHo is computed by the Kendall consistency coefficient (KCC) of blood oxygen level-dependent (BOLD) time series. ReHo has a good test–retest reliability (Zuo and Xing, 2014), and it can show the local features of cerebral activity. Higher ReHo values indicate higher coherence and centrality of local brain activity (Lv et al., 2018). ReHo is usually calculated in the low-frequency range, with low frequencies (0.01–0.08 Hz) being sensitive to cortical activity (Song et al., 2014). Many studies have found abnormal ReHo in patients with MDD, such as MDD with gastrointestinal symptoms (Yan et al., 2021a), melancholic MDD patients (Yan et al., 2021b), treatment-resistant depression (Guo et al., 2011b), and first-episode and treatment-naive depression (Guo et al., 2011a). Up to now, it is still unclear whether ReHo is related to various clinical indicators in MDD.

Previous studies separately report that patients with MDD have a diversity of brain structural and functional connectivity abnormalities, which are associated with manifold abnormal indicators. Nonetheless, there is an inconsistency with the results on abnormal functional synchronization of brain regions at rest in patients with MDD, and the interconnection with multifarious indicators remains unclear. Therefore, we collected fasting blood samples, HAMA, HAMD, SDSS, SSRS, SCSQ, EPQ, WCST, SCWT, RBANS, eye-tracking test, ERP and resting state MRI data of MDD patients and healthy subjects for comparison, and conducted correlation analysis with abnormal ReHo value of depression. This study aims to examine the alterations of brain functional synchronization in patients with MDD and their correlations with various indicators including biological, clinical, psychological, and cognitive indicators. We hypothesized that patients with MDD would exhibit abnormal ReHo in multiple brain regions, which were associated with clinical and cognitive parameters.

## Materials and methods

### Participants

Because of head movement and data deficiency, we excluded 4 patients with MDD and 3 HCs. Finally, 42 patients with MDD and 42 HCs were enrolled in the analysis. We recruited patients with MDD from the outpatient and inpatient departments of Foshan Third People’s Hospital, which are aged between 18 and 60 years old. According to the *Diagnostic and Statistical Manual of Mental Disorders, fifth edition* (*DSM*-5; American Psychiatric Association, 2013), the diagnosis was determined independently by two psychiatrists. All patients met the following inclusion criteria: (1) patients with first-episode or recurrent MDD; (2) right-handed; and (3) patients with first-episode MDD were drug-naïve and patients with recurrent MDD were drug-free for at least 2 weeks. Exclusion criteria were: (1) serious physical diseases especially organic cardiopathy or substance abuse; (2) other serious mental diseases, including schizophrenia, bipolar disorders, intellectual disability, and dementia; and (3) patients currently undergoing or preparing to undergo other clinical studies.

HCs were recruited from the local community through posters. They were excluded if they suffered from any medical and neurological disorders, psychotic symptoms, and substance abuse. All subjects obtained relevant information through a written informed consent and this study was approved by the Ethics Committee of Foshan Third People’s Hospital.

### Collection of related indicators

Twelve milliliters of peripheral venous blood was collected from all subjects at a fasting state. Blood lipid, thyroid hormone, cortisol, and uric acid were detected by the enzymatic method with the automatic biochemical analyzer. Electrocardiography (ECG) data were collected by a 12-lead ECG machine. HAMA and HAMD were used to evaluate emotional states. EPQ, SDSS, SSRS, and SCSQ were applied to evaluate the psychological status of the subjects. The RBANS, SCWT, and WCST were utilized to assess cognitive function. Eye movement analysis was performed using an eye movement analyzer, and event-related brain potentials were analyzed using an evoked potentiometer.

### Image capture and processing

Resting-state functional magnetic resonance imaging was performed using a 3.0 T GE scanner (GE 3.0 T Signa Pioneer). During image collection, subjects were required to keep quiet and still and stay awake with closing their eyes. Foam pads were used to reduce the head movement of the subjects, and soft earplugs were used to reduce the noise of the scanner. The parameter setting of repetition time (TR)/echo time (TE) in this study is 2000/30 ms, the number of layers is 36, the number of rows and columns in the MR image layer is 64*64 matrix, the flip Angle is 90°, the field of view (FOV) is 24 cm, the thickness of the exciting layer is 4 mm, no gap, and a total of 250 volumes (500 s).

Data Processing Assistant for Resting-State fMRI (DPARSF) software package (Chao-Gan and Yu-Feng, 2010) was used to preprocess the collected image data. Since the signal was unstable at the beginning of the collection and subjects needed to adapt to the environment, the first 10 time points of each subject were removed. Subjects with a maximum translation of no more than 2 mm in the X, Y, and Z axes and a maximum rotation of no more than 2° in each axis were included in the subsequent analysis. The 240 left volumes underwent slice timing and head motion correction. Then, the data of different subjects were registered to the standard MNI space using the echo plane imaging (EPI) template to solve the problem of brain morphology differences between different subjects and spatial location inconsistency during scanning. The data were resampled to 3*3*3 mm^3^ resolution. The acquired images were bandpass filtered (0.01~0.08 Hz) and linearly detrended.

### ReHo analysis

We performed ReHo analysis using the DPARSF toolbox to investigate the functional synchronization of spontaneous neural activity. ReHo describes the synchronization between a voxel and its neighboring voxel time series. The calculation formula of KCC has been stated in a previous study (Zang et al., 2004). In the voxel-based analysis method, the ReHo maps of subjects are obtained according to the KCC value of the time series of a given voxel and its nearest voxel (26 voxels). To reduce the influence of individual differences on KCC values, it is necessary to divide the KCC of each voxel by the average KCC of the whole brain to obtain a standardized ReHo profile. The generated imaging data were spatially smoothed and a Gaussian kernel with a full width of 4 mm was used to achieve a half-maximum value.

### Statistical analysis

A Chi-square test was used to analyze the difference in gender of patients with MDD and HCs. Two-sample *t*-tests were used to analyze the blood biochemical, ECG, psychological status, and cognitive indicators of the two groups. The significance level was set at *p* < 0.05.

For the voxel-based ReHo map, the differences between patients with MDD and HCs were compared by two-sample *t*-tests with education level, gender, age and mean framewise displacement (FD) as covariates. The significance level was corrected for multiple comparisons based on Gaussian Random Field (GRF) theory (voxel significance: *p* < 0.001, cluster significance: *p* < 0.05).

### Correlation analysis

Pearson/Spearman correlation analyses were performed to clarify the correlation between ReHo values and various indicators in the patients. *p* < 0.05 was considered as the significant threshold. The Bonferroni correction was performed for several dependent or independent statistical tests that were performed simultaneously.

## Results

### Participants and clinical baselines

There was no significant difference in years of education and gender between patients with MDD and HCs (Table 1). But there was a statistical difference in age (*p* = 0.001). There were significant differences in HAMD and HAMA (*p* < 0.001).

**Table 1:** Demography and clinical characteristics.

Variables	Patients	Controls	*p*-value
Age (years)	26.43 ± 10.79	35.14 ± 12.54	0.001^a^
Sex (male/female)	15/27	18/24	0.503^b^
Years of education (years)	13.48 ± 2.48	12.62 ± 3.72	0.218^a^
Height(cm)	163.64 ± 7.92	163.83 ± 7.85	0.912^a^
Weight(kg)	55.07 ± 11.06	59.88 ± 10.50	0.044^a^
HAMD	24.80 ± 7.22^c^	2.55 ± 3.54^c^	<0.001^a^
HAMA	16.60 ± 5.70^c^	2.03 ± 2.69^c^	<0.001^a^
TSH3UL(mIU/L)	1.65 ± 0.83^c^	2.17 ± 0.90^c^	0.226^a^
FT3(pmol/L)	4.38 ± 0.80^c^	4.80 ± 0.57^c^	0.006^a^
FT4(pmol/L)	14.64 ± 2.72^c^	14.55 ± 2.98^c^	0.893^a^
HR(times/min)	74.18 ± 11.67^d^	67.29 ± 10.22^c^	0.006^a^
QRS width(ms)	96.10 ± 11.60^d^	96.52 ± 11.36^c^	0.868^a^
PR interval(ms)	139.10 ± 16.73^d^	152.24 ± 19.20^c^	0.001^a^
QTc(ms)	371.40 ± 20.89^d^	394.95 ± 26.23^c^	<0.001^a^

HAMD, Hamilton Depression Rating Scale; HAMA, Hamilton Anxiety Rating Scale; TSH3UL, Thyroid Stimulating Hormone; FT3, Free Triiodothyronine; FT4, Free Thyroxine; HR, Heart Rate.

a The *p*-values were obtained by two sample *t*-tests.

b The *p*-value for sex distribution was obtained by a Chi-square test.

c *n* = 42.

d *n* = 40.

### Biological indexes differences between patients with MDD and HCs

In terms of thyroxine, we collected data from 42 individuals for each group of patients with MDD and HCs. There was a statistical difference in FT3 (*p* = 0.006), and no statistical difference was detected in the rest parameters (Table 1). Forty patients with MDD and 42 HCs had the ECG data. There were significant differences in HR (*p* = 0.006), PR interval (*p* = 0.001), and QTc (*p* < 0.001), except for QRS width (Table 1).

There were no statistically significant differences in TG, CHOL, HDL, and LDL between the two groups. In terms of cortisol, 41 data were collected from patients with MDD, and 42 data were collected from HCs. There was no statistically significant difference between the two groups. In terms of uric acid, 41 data were collected from patients with MDD or healthy subjects. There was no statistical difference between them (Supplementary Table S1).

### Psychological status differences between patients with MDD and HCs

As shown in Table 2, both 42 Patients with MDD and 42 HCs completed the psychological status assessment. There were significant differences in the scores of Extraversion (E; *p* < 0.001), Neuroticism (N; *p* < 0.001), and Lie (L; *p* = 0.038), except for Psychoticism (P) in the EPQ. There were significant statistical differences in the total scores of SDSS between patients with MDD and HCs (*p* < 0.001). In terms of SSS, there were significant differences in total scores (*p* < 0.001), objective support scores (*p* < 0.001), subjective support scores (*p* < 0.001), and utilization of support (*p* < 0.001) between the two groups. In terms of SCSQ, except for the total scores, there was a statistical difference between active coping (*p* < 0.001) and negative coping (*p* = 0.001).

**Table 2:** Comparison of patients and healthy controls in psychological status.

Variables	Patients (*n* = 42)	Controls (*n* = 42)	*p*-value
**EPQ**			
P	51.20 ± 8.19	47.23 ± 12.68	0.092^a^
E	40.16 ± 11.42	48.72 ± 13.93	0.003^a^
N	68.37 ± 9.19	45.08 ± 10.01	<0.001^a^
L	44.92 ± 11.30	56.93 ± 11.62	<0.001^a^
SDSS score	7.07 ± 2.42	0.02 ± 0.15	<0.001^a^
**SSS**			
Total score	28.60 ± 8.71	43.14 ± 9.33	<0.001^a^
Objective support score	7.45 ± 3.41	10.93 ± 2.85	<0.001^a^
Subjective support score	14.50 ± 5.23	23.36 ± 5.91	<0.001^a^
Utilization of support	6.64 ± 2.12	8.86 ± 2.05	<0.001^a^
**SCSQ**			
Total score	26.83 ± 8.42	29.90 ± 9.43	0.119^a^
Active coping	16.50 ± 6.19	22.90 ± 7.35	<0.001^a^
Negative coping	10.33 ± 4.24	7.00 ± 4.35	0.001^a^

EPQ, Eysenck Personality Questionnaire; P, Psychoticism; N, Neuroticism; E, Extraversion; L, Lie; SDSS, Social Disability Screening Schedule; SSS, Social Support Revalued Scale; SCSQ, Simplified Coping Style Questionnaire.

a The *p*-values were obtained by two sample *t*-tests.

### Cognitive status differences between patients with MDD and HCs

As shown in Table 3 and Supplementary Table S2, only Responses Answer (RA) showed significant differences between patients with MDD and HCs (*p* = 0.008) in the WCST examination, whereas Categories Completed (CC), Correct Responses (RC), Errors Responses (RE), Perseverative Responses (RP), and Perseverative Responses Errors (RPE) showed no significant differences. In the RBANS test, there was no significant difference between the two groups. In eye movement examination, there were statistical differences in the Number of Eye Fixation (NEF; *p* < 0.001), Responsive Search Score (RSS; *p* = 0.025), and Discriminant analysis (D; *p* = 0.004) between the two groups. In the ERP examination, there was no statistical difference between the two groups in N100 and P200, but there was a statistical difference in N200 (*p* = 0.038) and P300 (*p* = 0.012). To the reaction time of SCWT, there were significant differences in At, Bt, Ct, and C − 2B + 100 between patients with MDD and HCs (Table 4), but no significant differences in (C − B)/A, and in the error reaction.

**Table 3:** Comparison of patients and healthy controls in cognitive status.

Variables	Patients (*n* = 42)	Controls (*n* = 42)	*p*-value
**WCST**			
CC	5.00 ± 1.21	5.26 ± 1.23	0.328^a^
RA	46.05 ± 2.71	44.00 ± 4.02	0.008^a^
RC	34.55 ± 5.58	34.93 ± 3.58	0.711^a^
RE	11.50 ± 6.88	9.02 ± 6.21	0.087^a^
RP	3.57 ± 4.94	2.14 ± 3.33	0.124^a^
RPE	1.79 ± 2.85	0.81 ± 1.45	0.052^a^
**RBANS**			
Immediate memory	42.15 ± 10.40	42.83 ± 11.69	0.781^a^
Visuospatial/constructional	18.73 ± 2.16	17.90 ± 2.29	0.100^a^
Language	17.60 ± 4.43	18.57 ± 4.20	0.311^a^
Attention	60.60 ± 14.11	64.81 ± 16.09	0.212^a^
Delayed memory	48.33 ± 9.65	49.60 ± 10.66	0.574^a^
**EEM**			
NEF	21.11 ± 5.95	27.52 ± 4.32	<0.001^a^
RSS	3.71 ± 1.51	4.60 ± 1.62	0.025^a^
D	5.53 ± 1.40	4.47 ± 1.50	0.004^a^
**ERP**			
N100	103.73 ± 15.35	107.86 ± 34.86	0.508^a^
P200	174.53 ± 21.59	177.62 ± 23.66	0.545^a^
N200	230.21 ± 31.52	211.79 ± 44.69	0.038^a^
P300 (ms)	309.74 ± 24.20	287.10 ± 50.80	0.012^a^

WCST, Wisconsin card sorting test; CC, Categories Completed; RA, Responses Answer; RC, Correct Responses; RE, Errors Responses; RP, Perseverative Responses; RPE, Perseverative Responses Errors; RBANS, Repeatable Battery for the Assessment of Neuropsychological Status; EEM, Exploratory eye movement; NEF, number of eye fixation; RSS, responsive search score; D, Discriminant analysis; ERP, Event related potential.

a The p-values were obtained by two sample *t*-tests.

**Table 4:** Comparison of 41 patients and 42 healthy controls in SCWT.

Variables	Z-value	*p*-value
**Reaction time**		
At	−3.290	0.001^b^
Bt	−3.631	0.000^b^
Ct	−2.924	0.003^b^
(C − B)/A	−1.066	0.287^b^
C − 2B + 100	−2.519	0.012^b^

SCWT, Stroop color word test.

b The *p*-values were obtained by the Mann–Whitney *U*-test.

### ReHo: Group comparisons

**Figure 1:**
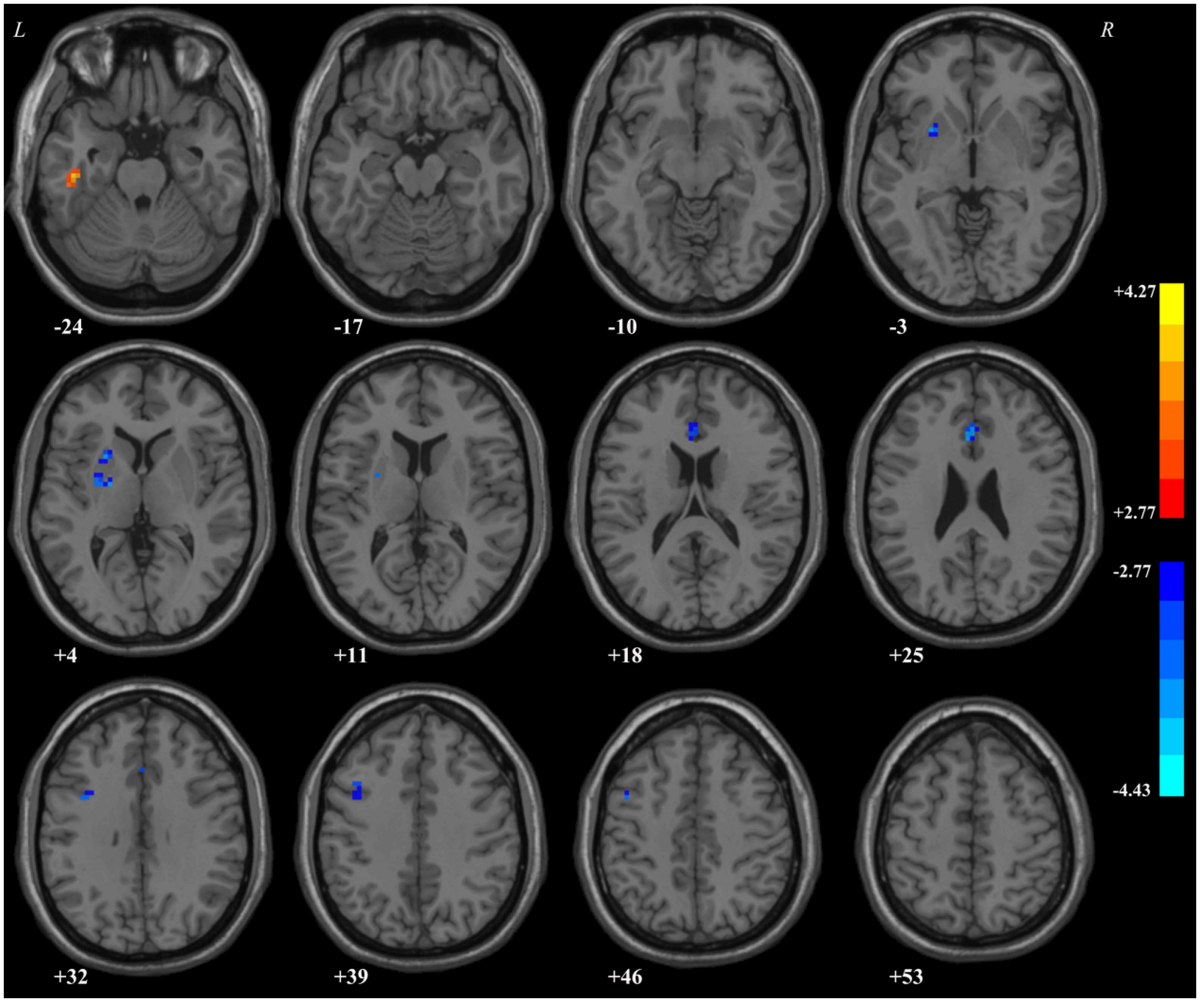
Regions with abnormal regional homogeneity values in the patients.

ReHo in the left ITG of patients with MDD significantly increased compared with that of HCs. By contrast, decreased ReHo values were found in the left putamen, anterior cingulate cortex (ACC), and precentral gyrus (Table 5; Figure 1).

**Table 5:** Regions with abnormal ReHo values in the patients.

Cluster location	Peak (MNI)			Number of voxels	*T-*value
	x	y	z		
Left putamen	−24	−6	3	52	−2.7740
Left anterior cingulate cortex	0	24	27	38	−2.7777
Left precentral gyrus	−45	6	48	32	−2.7984
Left inferior temporal gyrus	−45	−21	−24	23	3.9129

MNI, Montreal Neurological Institute; ReHo, regional homogeneity. The voxel size is 3*3*3 mm^3^.

### The correlations between ReHo values and symptoms and related factors

**Figure 2:**
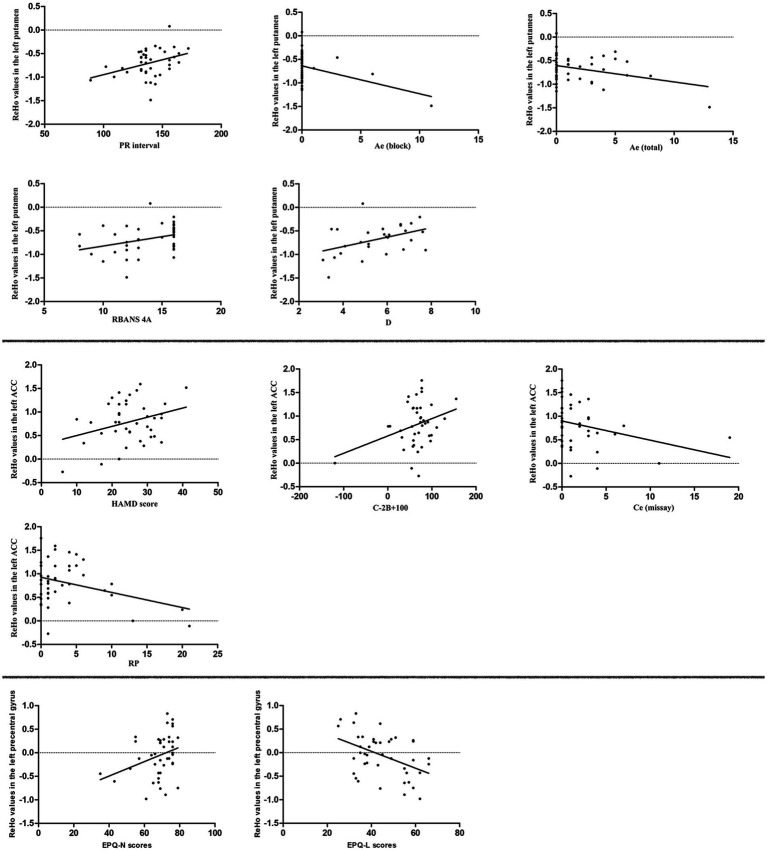
Pearson/Spearman correlation analyses showed that correlations between (1) the ReHo values in the left putamen and PR interval (r = 0.365, *p* = 0.021, df = 39); (2) the ReHo values in the left putamen and Ae (block; r = −0.397, *p* = 0.010, df = 40); (3) the ReHo values in the left putamen and Ae (total; r = −0.327, *p* = 0.037, df = 40); (4) the ReHo values in the left putamen and RBANS 4A (r = 0.334, *p* = 0.035, df = 39); (5) ReHo values in the left putamen and D (r = 0.428, *p* = 0.023, df = 27); (6) the ReHo values in the left ACC and HAMD score (r = 0.316, *p* = 0.047, df = 39); (7) the ReHo values in the left ACC and C − 2B + 100 (r = 0.326, *p* = 0.037, df = 40); (8) the ReHo values in the left ACC and Ce (missay; r = −0.315, *p* = 0.045, df = 40); (9) the ReHo values in the left ACC and RP (r = −0.346, *p* = 0.025, df = 41); (10) the ReHo values in the left precentral gyrus and EPQ-N score (r = 0.318, *p* = 0.040, df = 41); and (11) the ReHo values in the left precentral gyrus and EPQ-L score (r = −0.446, *p* = 0.003, df = 41). ReHo, regional homogeneity; ACC, anterior cingulate cortex; HAMD, Hamilton Depression Rating Scale; EPQ, Eysenck Personality Questionnaire.

As shown in Figure 2, the ReHo value of the left putamen was positively correlated with the PR interval of ECG (r = 0.365, *p* = 0.021), RBANS 4A (r = 0.334, *p* = 0.035), and D of eye movement examination (r = 0.428, *p* = 0.023), and negatively correlated with Ae (block; r = −0.397, *p* = 0.010) and Ae (total) of Stroop word color test (r = −0.327, *p* = 0.037) in the patients. The ReHo value of left ACC was positively correlated with HAMD score (r = 0.316, *p* = 0.047), C − 2B + 100 of Stroop word color test (r = 0.326, p = 0.037); and negatively correlated with Ce (Missay) of Stroop word color test (r = −0.315, *p* = 0.045) and RP of WCST (r = −0.346, p = 0.025) in the patients. The ReHo value of the left precentral gyrus was positively correlated with the N of EPQ (r = 0.318, *p* = 0.040) and negatively correlated with the L of EPQ (r = −0.446, *p* = 0.003) in the patients. However, no correlation could survive the Bonferroni correction. In addition, ReHo values of the left putamen, ACC, ITG, and precentral gyrus were not correlated with scores of HAMA, blood biochemistry, SSS, SDSS, and SCSQ in the patients.

## Discussion

This study was designed to detect the alterations of ReHo of patients with MDD and its relationship with a lot of clinical indicators. We observed notable differences between patients with MDD and HCs, with ReHo values increased in the left ITG; and decreased in the left putamen, ACC, and precentral gyrus. In the meanwhile, the ReHo values of left PG, putamen, and ACC are interconnected with multitudinous indicators in patients with MDD.

The results showed that abnormal ReHo values in patients with MDD were concentrated to the left side of the brain. Previous studies on the dominant cerebral hemisphere had shown that the left hemisphere had a dominant role in coordinating the hands to carry out complex movements and processing tools, as well as using language to communicate (Goodglass and Kaplan, 1963). Therefore, we speculated that the left cerebral hemisphere had a key role in MDD.

Brain network dysfunction is thought to underlie the cognitive and emotional abnormalities in MDD (Kaiser et al., 2015). Current researches have shown that MDD is caused by the interaction of three key networks (the central executive network, default mode network, and salience network) and the deficits in the functional connections between them and other brain regions (Culpepper, 2015). In this study, brain regions with abnormal ReHo values in patients with MDD were mainly involved in the salience network. The regional brain components of the salience network contain the dorsal ACC, putamen, anterior insula, and mid-cingulate, which mediate emotional regulation, monitoring for salient events, interoceptive awareness, and motivational behaviors (Yun and Kim, 2021).

Consistent with previous studies (Yang et al., 2015; Fu et al., 2018; Liu et al., 2021), the ReHo values of the left putamen were reduced in patients with MDD. Previous studies have revealed that the left putamen volume was smaller in patients with MDD than that in HCs, and the left putamen was smaller in melancholic MDD compared with non-melancholic MDD (Sachs-Ericsson et al., 2018), suggesting that abnormal spontaneous neural activity in the left putamen of patients with MDD has an anatomical basis. The results of correlation analysis showed that the ReHo values of the left putamen were positively correlated with D of the eye tracking test and RBANS 4A, and negatively correlated with Ae (block) and Ae (total) of SCWT. All of them are cognitive indicators, which are mainly related to attention and semantic retrieval. A study showed that the left anterior putamen operated in conjunction with classical language regionals in the dominant (left) hemisphere, directly related to semantic retrieval and comprehension by using the meta-analytic connectivity modeling (MACM) technique (Vinas-Guasch and Wu, 2017). In addition, other studies have shown that the left putamen is associated with the dorsal prefrontal cortex (DLPFC) by using probabilistic tractography, indicating that DLPFC may aid individuals to maintain mental health at both cognitive and emotional levels (Brosch et al., 2021). The putamen is an important node of the salience network, and DLPFC is an important component of the executive control network (Gunning et al., 2021). Therefore, reduced ReHo values in the left putamen may lead to dysfunctions of the salience network and cognitive impairments in MDD. The DLPFC played a role in goal-driven attention, working memory, task switching, problem-solving, planning, and novelty seeking (Jones and Graff-Radford, 2021). Therefore, abnormal ReHo value in the left putamen leading to cognitive dysfunction in patients with MDD might be directly affecting semantic retrieval and understanding, and further leading to cognitive dysfunction by affecting DLPFC.

As for the correlation between ReHo values and clinical indicators in patients with MDD, abnormal ReHo values of the left putamen were positively correlated with the PR interval. Valenza G et al. demonstrated that brain regions, such as the frontal gyrus, insula, paracingulate and cingulate cortex, lateral occipital cortex, and precuneus cortex, as well as subcortical structures (putamen, thalamus, globus pallidus, amygdala, hippocampus, brainstem, and right caudate nucleus) were involved in the regulation of ANS and mediated cardiovascular control (Valenza et al., 2017). Patients with ANS dysfunction can give rise to sympathoadrenal (SA) hyperactivity, which can cause elevated heart rates (Otte et al., 2005). Meanwhile, coupling with the decrease in parasympathetic tone may contribute to ventricular arrhythmias, and likely interpret the reason for higher cardiovascular mortality in patients with MDD and CVD (Halaris, 2017). Therefore, abnormal ReHo values in the left putamen may be involved in the regulation of ANS, and then affected the PR interval.

In line with a previous study (Xue et al., 2016), the ReHo values of the left ACC were decreased in patients with MDD compared with HCs. A VBM analysis found a decrease in gray matter volume in the left ACC of MDD compared with HCs (Chen et al., 2018). The ACC, a part of the neocortex, is involved in various cognitive functions such as misrecognition, emotional control, and adaptation to change (Allman et al., 2001; Gasquoine, 2013). ACC is involved in executive control in semantic processing through extensive connectivity with the sensory and motor cortices, and impaired connectivity with the ACC may lead to deficits in semantic performance (Zhao et al., 2017). In the present study, correlation analysis showed that the reduced ReHo values of left ACC were positively correlated with HAMD scores, C − 2B + 100 at reaction time in SCWT, and negatively correlated with Ce of SCWT and RP of WCST in the patients. Except for HAMD, they are cognitive indicators. RP is one of the best indicators of WCST to indicate cognitive flexibility (Heaton, 1981). SCWT tests the ability of individuals to overcome the occurrence of interference between two different dimensions of a stimulus through inhibitory control and selective attention mechanisms (Macleod, 1991). Previous studies have demonstrated that the DLPFC, ACC, and striatum are involved in SCWT tasks (Nee et al., 2007), and ACC is thought to monitor conflicts or errors during the SCWT tasks (Milham et al., 2001; Liu et al., 2006). Therefore, reduced ReHo values of the left ACC may contribute to cognitive impairment in MDD by directly affecting the salience network.

Decreased ReHo values were observed in the left precentral gyrus and were positively correlated with EPQ-N scores and negatively correlated with EPQ-L scores in the patients. Reduced ReHo values of the left precentral gyrus in patients with MDD have been found in the published literature (Zhang et al., 2021; Song et al., 2022). Previous studies have shown that patients with MDD who attempted suicide had fewer hemodynamic responses in the left precentral gyrus than patients with MDD without suicidal ideation and HCs (Tsujii et al., 2017). For EPQ, neuroticism has been identified as a risk factor for depression (Grav et al., 2012). Taking into account the higher score of the Lie subscale, the more stable and mature personality, Lie subscale is conducive to the development of behavior (Cao and Su, 2007).

The ITG plays an important role in visual object recognition, decision-making, and attentional impulse (Herath et al., 2001; Li and Kong, 2017). Some studies have shown that the left ITG is essential in lexical and phonological decision-making (Mechelli et al., 2003). In this study, the ReHo values of left ITG in patients with MDD increased, but no correlation with other indicators was observed. Kocsis K et al. found that the more severe depressive symptoms, the higher level of the asymmetry of ITG in patients with MDD (Kocsis et al., 2021). The results of a fractional Amplitude of Low Frequency Fluctuation (fALFF) study showed that the fALFF values of the left ITG were significantly increased in patients with MDD, and the fALFF values of the left ITG were correlated with the score of the Continuous Performance Test (CPT) second subtest (Huang et al., 2017). A voxel-based morphometry (VBM) study has shown a reduction in gray matter volume (GMV) in the right inferior temporal gyrus (Guo et al., 2014). Increased ReHo in the left ITG of patients with MDD may represent the presence of left–right hemisphere asymmetry, indicating that our results are consistent with those of previous studies.

Although there are valuable findings, there are still some limitations in this study. First, the sample size is relatively small. Second, the age of the two groups was mismatched, and mismatched age might have effects on our results although it was applied as a covariate in the analyses. Third, all correlations could not survive the Bonferroni correction although there were several correlations between abnormal ReHo and clinical and cognitive parameters in the patients. Thus, these results should be interpreted with caution. Finally, changes in ReHo values in patients with MDD after treatment were not tracked. Future studies with a view of the dynamic changes in ReHo values in patients with clinically cured MDD are needed to clarify whether the changes in ReHo values in patients with MDD are a trait phenomenon or a state marker.

Overall, this study is the first to evaluate the correlations between ReHo values and numerous indicators of MDD, including biological, clinical, psychological, and cognitive indicators. We found that the ReHo values were mainly significantly correlated with the cognitive indicators of patients with MDD, suggesting that the reduced ReHo values in brain regions of the salience network might be the underpinning of cognitive impairments in patients with MDD.

## Data availability statement

The raw data supporting the conclusions of this article will be made available by the authors, without undue reservation.

## Ethics statement

The studies involving human participants were reviewed and approved by the Ethics Committee of Foshan Third People’s Hospital. The patients/participants provided their written informed consent to participate in this study.

## Author contributions

YW, XL, and HY: methodology, data curation, formal analysis, and writing and editing. YO, WeW, WS, WC, YY, JL, WaW, HL, ZL, and XM: conceptualization and data curation. GX and WG: methodology, data curation, writing—review and editing, and funding acquisition. All authors contributed to the article and approved the submitted version.

## Funding

This study was supported by grants from the “The 14th Five-Year” Medical High-level Key Medical Specialty Development Project of Foshan (grant no. FSGSP145069), the project of Foshan Science and Technology Bureau (grant no. 2020001005608), and National Natural Science Foundation of China (grant no. 82171508).

## Conflict of interest

The authors declare that the research was conducted in the absence of any commercial or financial relationships that could be construed as a potential conflict of interest.

## Publisher’s note

All claims expressed in this article are solely those of the authors and do not necessarily represent those of their affiliated organizations, or those of the publisher, the editors and the reviewers. Any product that may be evaluated in this article, or claim that may be made by its manufacturer, is not guaranteed or endorsed by the publisher.

## Supplementary material

The Supplementary material for this article can be found online at: https://www.frontiersin.org/articles/10.3389/fpsyg.2022.1090181/full#supplementary-material
