# Supplementary material for: Multiple examinations indicated associations between abnormal regional homogeneity and cognitive dysfunction in major depressive disorder
Source: Front Psychol. 2023 Jan 26;13:1090181. doi: 10.3389/fpsyg.2022.1090181 (PMC9909210; doi:10.3389/fpsyg.2022.1090181)
Supplement: Supplementary file 1 [file Table_1.docx]

Table S1. Blood biochemical indicators

| Variables | Patients (n = 42) | Controls (n = 41) | *p*-value |
| --- | --- | --- | --- |
| TG（mmol/L） | 0.98±0.65 | 1.09±0.49 | 0.351^a^ |
| CHOL（mmol/L） | 4.38±1.05 | 4.65±0.72 | 0.163^a^ |
| HDL（mmol/L） | 1.32±0.39 | 1.25±0.30 | 0.333^a^ |
| LDL（mmol/L） | 2.41±0.84 | 2.62±0.58 | 0.199^a^ |
| Cortisol（nmol/L） | 331.91±126.50 | 304.78±109.43 | 0.299^a^ |
| Uric acid（μmol/L) | 334.80±100.83 | 352.30±101.13 | 0.435^a^ |

Blood lipid measurements included 42 MDD patients and 41 healthy controls.TG=Triglyceride; CHOL=Cholesterol; HDL=High Density Lipoprotein; LDL=Low Density Lipoprotein

^a^ The *p*-values were obtained by two sample *t*-tests.
