# Supplementary material for: Multiple examinations indicated associations between abnormal regional homogeneity and cognitive dysfunction in major depressive disorder
Source: Front Psychol. 2023 Jan 26;13:1090181. doi: 10.3389/fpsyg.2022.1090181 (PMC9909210; doi:10.3389/fpsyg.2022.1090181)
Supplement: Supplementary file 2 [file Table_2.docx]

Table S2. The Comparison of 41 patients with MDD and 42 healthy controls in SCWT

| Variables | Z-value | *p*-value |
| --- | --- | --- |
| Error reaction |  |  |
| Ae(missay) | -1.556 | 0.120^b^ |
| Ae(correction) | -0.105 | 0.916^b^ |
| Ae(block) | -0.105 | 0.623^b^ |
| Ae(total) | -0.472 | 0.637^b^ |
| Be(missay) | -0.455 | 0.649^b^ |
| Be(correction) | -1.222 | 0.222^b^ |
| Be(block) | -0.612 | 0.541^b^ |
| Be(total) | -0.594 | 0.541^b^ |
| Ce(missay) | -1.093 | 0.274^b^ |
| Ce(correction) | -0.300 | 0.764^b^ |
| Ce(block) | -0.070 | 0.944^b^ |
| Ce(total) | -0.151 | 0.880^b^ |
| (C-B)/A | -0.506 | 0.613^b^ |
| C-2B+100 | -0.554 | 0.579^b^ |

^b^ The p-values were obtained by Mann-Whitney U test.
